# Supplementary material for: Adversarial AI applied to cross-user inter-domain and intra-domain adaptation in human activity recognition using wireless signals
Source: PLoS One. 2024 Apr 18;19(4):e0298888. doi: 10.1371/journal.pone.0298888 (PMC11025916; doi:10.1371/journal.pone.0298888)
Supplement: Attachment — Submitted filename: Response.docx [file pone.0298888.s001.docx]

1^st^ January 2024

Dear PLoS ONE Editor

**Re: PLOS ONE Decision: [PONE-D-23-32286]**

Thanks for your decision email. This letter details our revisions to the manuscript that take into account the reviewers’ comments. The comments are quoted in full; our comments are in blue.

**Journal Requirements:**

- Please ensure that your manuscript meets PLOS ONE's style requirements, including those for file naming.

Changes have been made in the previously submitted document regarding PLOS ONE's style requirements. Please see the track changes.

- Please note that PLOS ONE has specific guidelines on code sharing for submissions in which author-generated code underpins the findings in the manuscript. In these cases, all author-generated code must be made available without restrictions upon publication of the work. Please review our guidelines at https://journals.plos.org/plosone/s/materials-and-software-sharing#loc-sharing-code and ensure that your code is shared in a way that follows best practice and facilitates reproducibility and reuse.

Code for the proposed model is available in a GitHub repository in open access at: https://github.com/DASAN-MMD/Code. A link to the repository is provided in the data and code availability section of the revised version.

- We note you have included a table to which you do not refer in the text of your manuscript. Please ensure that you refer to Table 1-10 in your text; if accepted, production will need this reference to link the reader to the Table

Many thanks for reminding us about this. All the tables have been referred in the text in the revised version. Please see the track changes.

**Reviewer #1**:

- The paper's abstract needs further revision. The authors should shorten the abstract in a precise way.

There are many changes and removal of content from abstract to summarise it properly. Please see the revised version.

- The main contributions should be summarized one by one in the introduction.

Thanks for your suggestion. Our main contributions have been summarised in the end of the introduction. Please see the revised version.

- In related works, the authors summarize previous literatures instead of only listing them. On this basis, the novelty should be highlighted by presenting what has been previously explored or not.

Many thanks for pointing out this area. We have discarded the irrelevant explanation and listed the previous work in the revised version.

- The authors should place the related work section after the introduction section.

Changes have been made as per the suggestion.

- In particular, I strongly recommend the authors to restructure the tables and enlarge the font size, so as to make them more clear.

Thanks for your recommendation. It was difficult to enlarge the font size in previous submission as the tables were going out of the page margin. To do that, we have split each of the old tables into two and enlarged their font.

- I also recommend the authors to refer to related activity recognition literatures:10.1016/j.knosys.2023.110789; 10.1109/TKDE.2023.3277839; 10.1109/JBHI.2023.3275438; 10.1109/TII.2023.3315773.

Thanks for referring to some of the valuable content in the similar area. We went through this content, in fact some of them are cited in our related work session.

**Reviewer #2**:

- While the introduction part presents basic background information and concepts relevant to this study, a brief summary of the primary contributions at the outset would help understand the scholar value and academic impact of the research.

Thanks for your suggestion. Our main contributions have been summarised in the end of the introduction. Please see the revised version.

- The problem definition section of the manuscript illustrates the challenges inherent in this research area, but the formulation of these issues lacks precision and clarity. A clearer definition of the problem is needed to be provided for subsequent proposed methods and experiments.

Thanks for pointing out this. Though, in previous submission we tried to make the problem statement very simple for the reader, but we agree it’s a bit vague. We have made several changes in the problem definition in our revised version. Please see the track changes.

- For the proposed adversarial AI model, firstly, the theoretical foundations of the model are not well developed and a more robust theoretical framework is needed to support the integrity of the model. Also, there is a lack of innovative approaches and unique contributions that are currently existing in the field of adversarial AI. While the network structure employed in the model is common for a variety of inputs such as images, it would be better to have explanations on specific designs for handling WiFi CSI inputs.

Thanks for your comments. Additional text has been added related to specific designs for handling WiFi CSI inputs. Each module of the model has also been elaborated with mathematical expressions to provide more clarity. We completely agree with the statement that the proposed model is very common for a variety of image inputs but it’s a robust and simple model suitable for the analysis of global and sub-domain alignment using wireless sensing, which is our key contribution and its acceptability on CSI Image data for cross-user domain shifting task is a new initiative in this field. We have also shown key findings on a public CSI dataset using specific pre-processing steps. In summary, our model is not going to revolutionize this entire field, but it opens door for many key challenges in this field as future directions, such as investigating combined cross-environmental and cross-user features to make the WiFi sensing more appealing and applicable.

- The figures used in this paper can be better structured and illustrated.

We have improved figure structure. See the revised version.

Yours sincerely

**Thomas W Kelsey PhD, FRSM, MISFP**
